# Recovery Rate of Children From Pneumonia and Its Predictors in Ethiopia: A Systematic Review and Meta‐Analysis

**DOI:** 10.1002/hsr2.71127

**Published:** 2025-09-01

**Authors:** Temesgen Gebeyehu Wondmeneh, Abduhakm Hara Hadato

**Affiliations:** ^1^ Department of Public Health, College of Medical and Health Science Samara University Semera Afar Ethiopia

**Keywords:** children, Ethiopia, pneumonia, recovery rate

## Abstract

**Background:**

Sub‐Saharan Africa accounts for the majority of child pneumonia mortality and morbidity. The pooled recovery rate of children from pneumonia and its predictors is not well known in Ethiopia.

**Aim:**

The aim of this systematic review and meta‐analysis is to determine the pooled recovery rate of children from pneumonia and its predictors in Ethiopia.

**Methods:**

The major databases used to search articles were Web of Science, Science Direct, PubMed, Google Scholar, and African journals online. The data were extracted independently from eligible primary studies using a standardized spreadsheet. The quality of the included studies was assessed using the Newcastle–Ottawa scale critical appraisal checklist for the cohort study. The pooled effect size with a 95% CI was estimated by the random‐effects model of meta‐analysis. The amount of heterogeneity across the studies was assessed by *I* ^2^. A sensitivity analysis was conducted.

**Results:**

In this systematic review and meta‐analysis, 6173 children with pneumonia were included; out of these, 4871 had recovered. The pooled recovery rate of children from pneumonia was 17.7 (95% CI: 14.61–20.79) per 100 children per day. Children who lived in rural areas (AHR = 0.81, 95% CI: 0.74–0.88), stunted children (AHR = 0.77, 95% CI: 0.56–0.99), children with dangerous signs (AHR = 0.78, 95% CI: 0.66–0.9), not fully vaccinated children (AHR = 0.55, 95% CI: 0.11–0.99), comorbid children (AHR = 0.57, 95% CI: 0.48–0.65), and children with a history of respiratory infection (AHR = 0.86, 95% CI: 0.76–0.96) had a lower recovery rate from pneumonia.

**Conclusion:**

In the current study, the recovery rate of children from pneumonia was 17.7 per 100 child‐days. Children living in rural areas recovered more slowly. Healthcare providers should give special attention at admission for screening of children with stunted, danger signs, not fully vaccinated, comorbid, and histories of respiratory tract infection.

**Abbreviations**
AHR: adjusted hazard ratio
CI: confidence interval
Fig: figure
S: supplementary

## 1 Background

Pneumonia is a respiratory‐acute lung infection. The most common symptoms of pneumonia are coughing, trouble breathing, and fever [1]. Pneumonia is the leading infectious cause of mortality for children globally. About 740,180 children under the age of 5 in 2019 were killed by pneumonia, contributing 14% of all deaths of children under 5 years old [2]. New estimates from the Global Burden of Diseases (GBD) showed that the number of deaths of children from pneumonia was 693,000 in 2019 and 502,000 in 2021. This 28% reduction over 3 years is due to the impact of COVID‐19 restrictions and underscores the lifesaving power of preventing the spread of infection [3]. The mortality rate of pneumonia is lower in developed countries, being less than 1 per 1000 per year [4]. In a study of Nepal, the median time till recovery was 49 h. Treatment failure was experienced by 35% of the children [5]. In Ethiopia, a study showed that the incidence of recovery from severe pneumonia among children aged 2–59 months was 92.3%, with a median time of recovery of 4 days [6]. In a study of Jimma district in Ethiopia, about 83.91% of under‐five children improved from severe pneumonia [7].

The changing etiological profile of pneumonia can influence the recovery rate [8]. Reductions in pneumonia mortality are reliant upon the strengthening of basic health care, particularly immunization, and the improvement of service utilization [9]. Preventing and treating pneumonia required improving facility preparedness, providing high‐quality care, and scaling up demand to intensify efforts [10]. The risk factors for pneumonia mortality in young children are a delay in seeking appropriate care and inaccessibility to multiple treatment sources [11]. Careful diagnosis and management of danger signs like hypoxia are important for the recovery of children from pneumonia [12]. Mothers and other caregivers should recognize the symptoms and danger signs of pneumonia to provide timely treatment, which is essential for the children's survival [13]. Accurate recognition of the child with severe pneumonia, supported by a mechanism that allows rapid referral to a facility with parenteral antibiotics and oxygen, is critical but currently inadequate in resource‐limited settings [14]. Improving housing quality, and improving early childhood nutrition may reduce pneumonia [15]. The risk of death from childhood pneumonia dramatically increases with the severity of malnutrition [16]. In children with pneumonia, stunting prolongs the duration of recovery and raises the risk of treatment failure [17]. The risk factors for host‐related community‐acquired pneumonia are malnutrition, young age, comorbidities (congenital heart and lung disease), low birth weight, a lack of breastfeeding, an incomplete series of vaccinations, and viral respiratory infections [18].

Understanding the pneumonia recovery rate provides insight about the burden of the disease on the healthcare system and caregivers, as well as the information that can be used to guide health interventions. The recovery rate reflects the quality of the available health services at the health institution. Even though there were primary studies at district levels in Ethiopia, there is no study regarding the pooled recovery rate of children from severe pneumonia and its determinants at the national level. The study will enable the Ethiopian government to mobilize resources for the health care needs of the children for the intervention of childhood pneumonia. This may be used to enhance the recovery rate of children, thereby ensuring adequate management and reducing the risk of death. Therefore, the aim of this study was to determine the pooled recovery rate of children from pneumonia and its determinants in Ethiopia.

## 2 Methods

### 2.1 Study Area

The study was conducted in Ethiopia. The country is divided into two administrative cities (Addis Ababa and Dire Dawa) and nine regional states (Tigray, Afar, Amhara, Oromia, Somalia, Benishangul, SNNPR, Gambela, and Harari). Benishangul, Gambela, Somalia, and Afar were considered as developing regions. Primary, secondary, and tertiary levels of care are the three tiers of Ethiopia's current healthcare delivery system. Primary hospitals, health centers, and community‐based health posts serve as the primary level of care. The secondary healthcare system consisted of a general hospital, which functions as a referral center for primary hospitals. The tertiary healthcare system includes a specialized hospital and a referral center for general hospitals.

### 2.2 Protocol and Report

This systematic review and meta‐analysis protocol was recorded in the International Prospective Register of Systematic Review (PROSPERO) with an identification number of CRD42024523582. This systematic review and meta‐analysis were reported based on the Preferred Reporting Items for Systematic Reviews and Meta‐Analyses (PRISMA) guideline [19] (Supporting Information S1).

### 2.3 Searching Strategies

Major databases used for searching were Web of Science, Science Direct, PubMed, Google Scholar, and African Journals Online. Additional searches were conducted using reference lists of included studies and Ethiopian higher institution repositories. The keywords and phrases used for searching were “recovery rate”, children, pneumonia, and Ethiopia. The Boolean operator “AND” was used to combine recovery rate, children, pneumonia, and Ethiopia. For each of these terms or phrases, all fields, any fields, or all words, depending on the database‐adapted field, were used to do sensitive searching and find all relevant articles. The search was guided by population (children), condition (pneumonia), outcome (recovery rate), and context (Ethiopia) (PCOCo). The initial search was conducted from March 24, 2024, to March 29, 2024, and was updated on October 10–13, 2024. Detailed search strategies were provided in Supporting Information S2.

### 2.4 Study Selection

Articles identified in various databases were exported to Endnote X8.1 software, and duplicated articles were excluded. The remaining articles were screened based on the articles' titles and abstracts in relation to this systematic review's objective. The full‐text articles that were considered essential were retrieved after reviewing the titles and abstracts of the articles. Two authors independently evaluated the eligibility of full‐text articles. Scientific consensus was used to settle disagreements between the two authors. After full‐text evaluation, eligible articles were found and included in the final meta‐analysis.

### 2.5 Inclusion and Exclusion Criteria

To establish inclusion and exclusion criteria, the population, intervention, comparator, and outcome (PICO) framework technique was used, which mainly involves population, outcome, and context (POCo) questions for the recovery rate of children from pneumonia.

#### 2.5.1 Inclusion Criteria

- The study population included all children.
- The case for this systematic review was the recovery rate of children from pneumonia, which was reported in the primary study as the incidence rate of recovery of children per person‐time.
- Studies conducted in Ethiopia.
- Observational studies, such as retrospective and prospective cohort studies, that reported the recovery rate of children from pneumonia.
- Published and unpublished full‐text articles.

#### 2.5.2 Exclusion Criteria

- Studies that did not report the outcome (recovery rate) as an incidence rate per person per time.
- Articles lacking the full text were excluded after contacting the corresponding authors by email at least twice.
- Review articles, commentaries, and editorials were excluded.

### 2.6 Outcome Measurement

The main outcome of interest was the recovery rate of children from pneumonia, which is reported in the primary article as the incidence rate of recovery of children per person‐time (child‐days). Two parameters were required to calculate the outcome: the number of children recovered from pneumonia and the person's time (child days). It was computed by dividing the number of children who recovered from pneumonia by the total person‐time (child‐days).

### 2.7 Data Extraction

Two authors independently extracted the data from the included studies. During data extraction, scientific consensus was used to resolve the disagreements between authors. Using a predefined data extraction format developed from Microsoft Excel, the following data were extracted: name of the first author, publication date, study periods, study region, study design, sample size, persons‐time (child days), number of children recovered from pneumonia, and incidence rate (Supporting Information S3).

### 2.8 Quality Assessment

The qualities of each included study were independently appraised by two authors using the Newcastle–Ottawa scale critical appraisal checklist for the cohort study [20]. Any disagreements between the two authors were resolved through discussion and scientific consensus. The Newcastle–Ottawa Quality Assessment Form for Cohort Studies consisted of three domain categories: selection (representativeness of the exposed cohort and selection of the nonexposed cohort, as well as ascertainment of exposure and outcome of interest at the start of the study), comparability, and outcome (assessment of outcome, long enough follow‐up for the occurrence of outcome, and adequacy of follow‐up). The quality scores of the included studies were classified as low quality when the quality of the included studies was less than 50% and good quality when the quality of the included studies was at least 50%. Only good‐quality articles were included in the final meta‐analysis.

### 2.9 Data Synthesis and Statistical Analysis

The extracted data from the Microsoft Excel spreadsheet was imported into STATA version 15 for additional statistical analysis. The pooled incidence rate of recovery with a 95% CI was estimated by the random‐effects model of meta‐analysis [21]. The pooled incidence rate of recovery was reported as 100 children per day. Heterogeneity across the studies was assessed by the *I* ^2^ statistic, where 25%, 50%, and 75% represent low, moderate, and high heterogeneities, respectively [22]. The existence of publication bias was checked by the funnel plot and Egger's tests [23, 24]. Trim and fill analyses were employed to find the missing studies in the event that publication bias was present. A sensitivity analysis was performed to examine the effect of a single study on the overall pooled incidence rate of recovery [25]. Subgroup analysis was conducted based on region, study design, and length of follow‐up. The pooled adjusted hazard ratios (AHR) were used to determine the association between the incidence rate of recovery and its associated risk factors. Two‐tailed tests are used to determine the significance level at 5%. Finally, the results of the meta‐analysis were presented in forest plots and tables, accompanied by textual or written explanations.

### 2.10 Ethical Approval

Since the systematic review and meta‐analysis were used in previously published primary studies, ethical approval was not needed.

## 3 Results

### 3.1 Search Results

**Figure 1:**
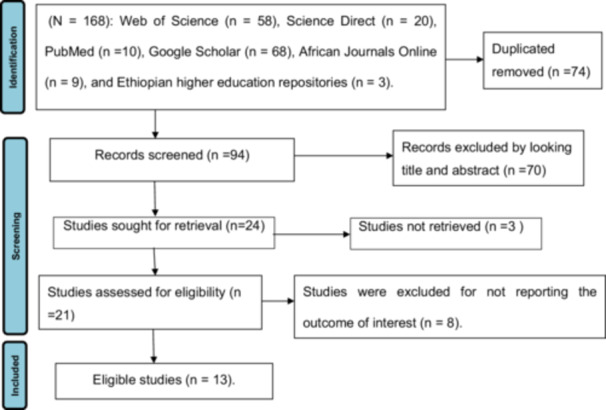
Shows the PRISMA flow chart for the selection of studies for systematic review.

The initial search result provided a total of 168 articles using the following various databases. Seventy‐four duplicated articles were removed by Endnote X8.1 software. After reviewing titles and abstracts, 70 irrelevant articles that did not align with the objective of the study were excluded. The remaining 24 articles were subjected to full‐text evaluation. After full‐text evaluation, eleven articles were excluded for various reasons. Finally, 13 eligible articles were found (Figure 1).

## 4 Characteristics of Included Studies

In the final meta‐analysis, a total of 13 eligible studies were included. Most of the included studies were conducted in the Amhara region. Three studies were conducted in Southern Nations Nationalities and Peoples (SNNP), and two in Oromia. There was a single study in each of the Beshangul Gumuz and Addis Ababa administrative cities. The majority of studies were retrospective. The total person‐time was 29,411 children per day. The minimum person‐time was 100 children per day [26], and the maximum person‐time was 5134 children per day [27]. The maximum incidence rate of recovery was 26.71 per 100 children per day [28], and the minimum incidence rate of recovery was 9.6 per 100 children per day [29]. The study period ranged from 2015 to 2022 (Table 1).

**Table 1:** Study characteristics.

Authors	Study year	Study region	Study design	Sample size	Cases	Person‐time (children‐day)	Incidence rate per 100 child‐day
Kassaw et al. [29]	2021–2022	Amhara	Prospective	580	451	4716	9.6
Mengist et al. [37]	2016–2018	Amhara	Retrospective	352	313	1923	16.25
Dinku et al. [35]	2016–2020	Beshangul Gumuz	Retrospective	515	488	2478	19.69
Sinishaw et al. [26]	2018–2020	Addis Ababa	Retrospective	388	11.5	100	11.5
Tirore et al. [33]	2017–2020	SNNP	Retrospective	280	260	1076	24.16
Assfaw et al. [36]	2015–2020	Amhara	Retrospective	330	120	889	13.5
Tamirat et al.[28]	2018–2020	Amhara	Retrospective	701	688	2567	26.71
Bethelhem [38]	2019–2021	Amhara	Retrospective	587	427	2863	14.53
Genie et al. [27]	2022	SNNP	Prospective	791	641	5134	12.5
Dinka et al. [30]	2017–2022	Oromia	Retrospective	376	356	1599	22.26
Teferi et al. [31]	2021	Amhara	Prospective	270	205	1002	20.45
Kebede et al. [34]	2019–2021	SNNP	Retrospective	591	541	2245	24.1
Wake [32]	2021–2022	Oromia	Retrospective	412	369	2819	13.1

*Note:* Cases: recovered children from pneumonia.

Abbreviation: SNNP, southern nation nationality people.

### 4.1 Quality of Included Studies

Studies with a quality score of five or higher were considered to have a high quality score. The quality scores of all included studies were 9 out of 9 (100%). Thus, all 13 included studies had a high quality score (Supporting Information S4).

### 4.2 Pooled Recovery Rate of Children From Pneumonia

**Figure 2:**
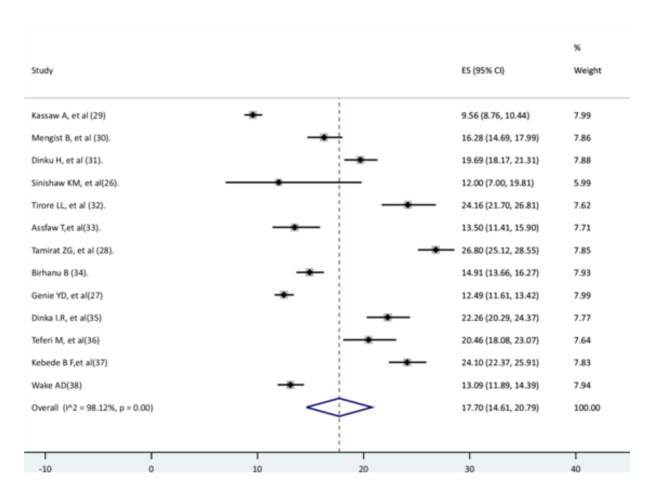
Pooled recovery rate of children from pneumonia.

A total of 6173 pneumonia cases were identified; out of these, 4871 were recovered. The total person‐time was 29,411 children‐days. Based on the random‐effects model, the pooled recovery rate of children from pneumonia was 17.7 (95% CI: 14.61–20.79) per 100 children per day. There was a significant high heterogeneity between (*I* ^2 ^= 98.12%, *p*< 0.001) (Figure 2).

### 4.3 Subgroup Analysis

The highest recovery rate was observed in the Southern Nation Nationality People (SNNP) and Benshangul Gumuz at 20 per 100 children per day, while the lowest recovery rate was observed in Addis Ababa administrative city at 12 per 100 children per day. High heterogeneity was observed in the Amhara region (*I* ^2^ = 98.6%, *p* < 0.001). Using retrospective study designs, the recovery rate of children from pneumonia was 19 per 100 children per day (95% CI: 16–22), and significant heterogeneity was exhibited between studies (*I* ^2 ^= 96.9%, *p*< 0.001). The recovery rate was 12 per 100 children per day (95% CI: 10–15) for median follow‐up periods longer than 4 (Table 2).

**Table 2:** Subgroup analysis.

Variables	Categories	Number of study	Sample size	Recovery rate per 100 children‐day	*I* ^2^, *p* value
Region	Amhara	6	2820	17 (95% CI: 12–22)	98.6%, < 0.001
	Addis Ababa	1	388	12 (95% CI: 7–20)	—
	Oromia	2	788	16 (95% CI: 15–17)	—
	SNNP	3	1662	20 (95% CI: 11–29)	—
	Benshangul Gumuz	1	515	20 (95% CI: 18–21)	—
Study design	Retrospective	10	4532	19 (95% CI: 16–22)	96.9%, < 0.001
	Prospective	3	1641	14 (95% CI: 10–18)	—
Median recovery time	> 4 days	5	2758	12 (95% CI: 10–15)	92.78%, < 0.001
	≤ 4 days	8	3415	21 (95% CI: 18–24)	95%, < 0.001

*Note:* Dash (—) indicates none.

Abbreviation: SNNP, southern nation nationality people.

### 4.4 Publication Bias

**Figure 3:**
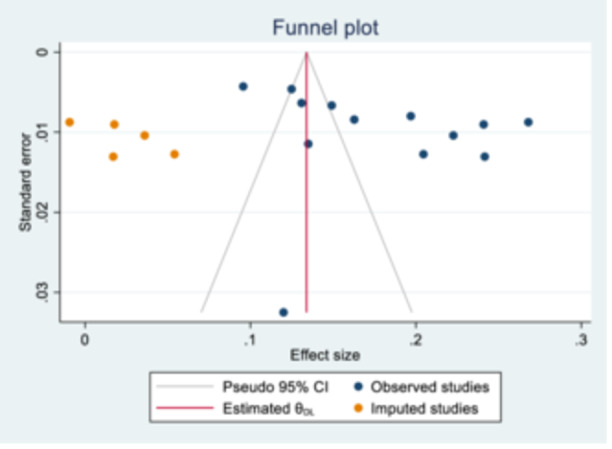
Symmetrical funnel plot by meta‐trim and fill plot.

Inspection of the funnel plot showed an asymmetric funnel plot. The Egger's test also confirmed the presence of publication bias (bias = 11.1 (95% CI: 2.2–20), *p* = 0.019). Due to the existence of publication bias, a trim‐and‐fill analysis was used to identify five missed studies. A combined number of missed and observed studies yielded a total of 18 studies. The pooled effect size decreased from 17.7% (95% CI: 14.6–20.8) in the observed studies to 13.4% (95% CI: 10–16.8) in the combined studies. A trim and fill plot incorporating the missed studies and then mirroring them on the opposite side to estimate the unbiased pooled effect size is shown in Figure 3.

### 4.5 Sensitivity Analysis

**Figure 4:**
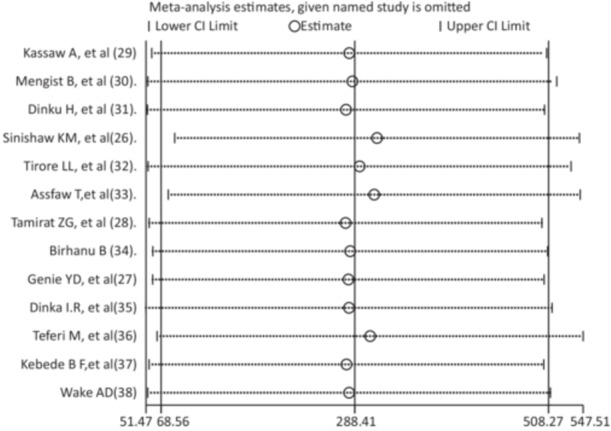
Sensitivity analysis of the recovery rate of children.

The sensitivity analysis revealed a single study did not have any effect on the overall estimate of recovery rate. The results of the sensitivity analysis were fairly stable (Figure 4).

## 5 Determinants of the Recovery Rate of Children From Pneumonia

### 5.1 The Association Between Rural Residence and Recovery Rate

**Figure 5:**
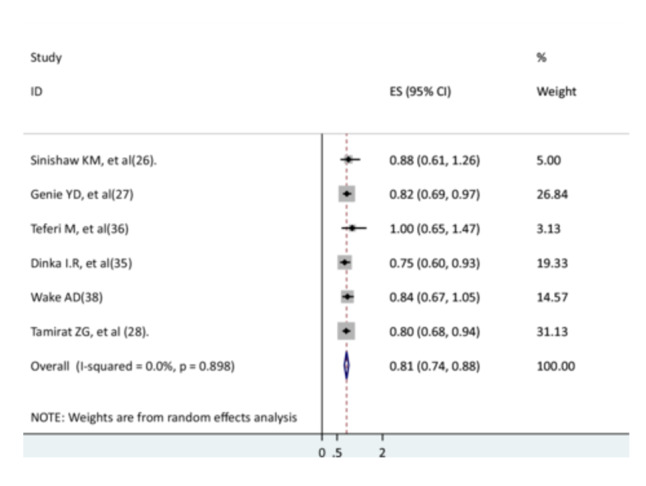
The association between rural residence and recovery of children from pneumonia.

Six studies [26, 27, 28, 30, 31, 32] were found to determine the association between rural residence and the recovery rate of children from pneumonia. Three studies [27, 28, 30] had a significant association with recovery rate, while the other three did not have such a significant association [26, 31, 32]. The results of this meta‐analysis showed that rural children were 19% less likely to recover from pneumonia than urban children (AHR = 0.81; 95% CI: 0.74–0.88), with no heterogeneity (Figure 5).

### 5.2 The Association Between Exclusive Breastfeeding and Recovery Rate

**Figure 6:**
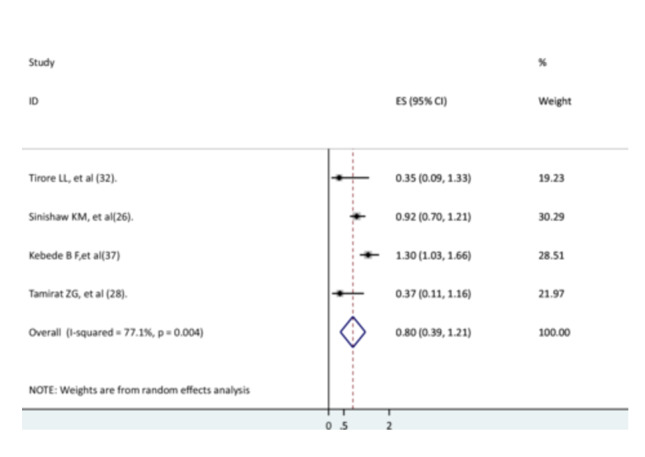
The association between exclusive breastfeeding and recovery rate.

Four studies [26, 28, 33, 34] were used to determine the association between nonexclusive breastfeeding and the recovery rate of children from pneumonia. The findings of the three studies [26, 28, 33] did not show a significant association with the recovery rate, while the findings of one study showed a significant association [34]. This meta‐analysis result indicated that children without exclusive breastfeeding did not have a significantly different recovery rate compared to those with exclusive breastfeeding (AHR = 0.8, 95% CI: 0.39–1.21) in the presence of high heterogeneity (Figure 6).

### 5.3 The Association Between Nutritional Status and Recovery Rate

**Figure 7:**
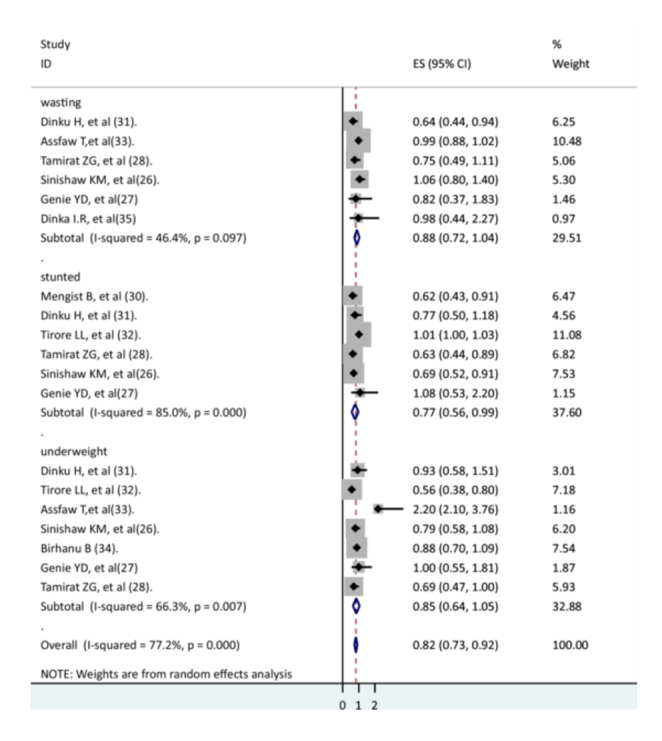
The association between nutritional status and recovery rate.

The association of wasting [26, 27, 28, 30, 35, 36], stunting [26, 27, 28, 33, 35, 37], and underweight [26, 27, 28, 33, 35, 36, 38] with recovery rate was determined in this systematic review and meta‐analysis. In the present systematic review and meta‐analysis, the recovery rate of children from pneumonia did not show a significant association with wasting (AHR = 0.88, 95% CI: 0.72–1.04) or underweight (AHR = 0.85, 95% CI: 0.64–1.05); however, the recovery rate of children from pneumonia was lower for those with stunting (AHR = 0.77, 95% CI: 0.56–0.99). There were heterogeneities between studies (Figure 7).

### 5.4 The Association Between Anemia and Recovery Rate

**Figure 8:**
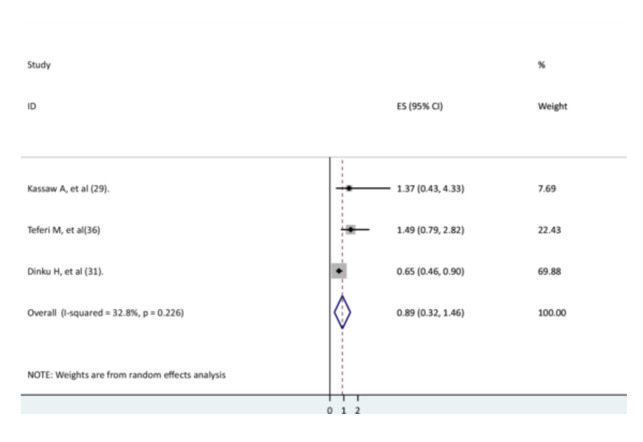
The association between anemia and recovery rate.

Three studies examined the relationship between anemia and recovery rate [29, 31, 35]. One study found that children who were anemic had a lower rate of recovery from pneumonia [35], whereas another two studies found no significant relationship [29, 31]. The current meta‐analysis found that anemic children did not have a significant recovery from pneumonia than non‐anemic children (AHR = 0.89, 95% CI: 0.32–1.46). There was low heterogeneity between studies (Figure 8).

### 5.5 The Association Between Danger Signs and Recovery Rate

**Figure 9:**
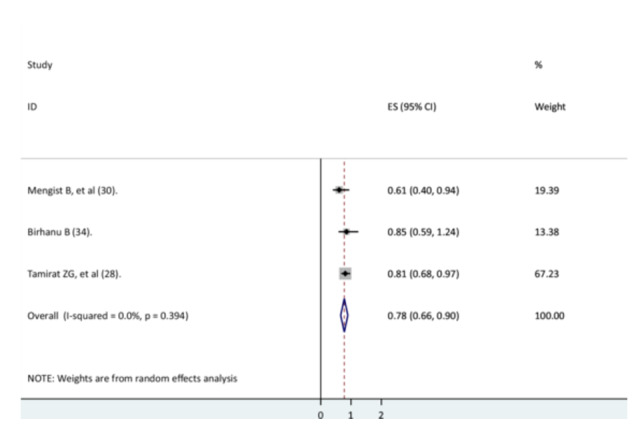
The association between danger signs and recovery rate.

Three studies [28, 37, 38] were used to investigate the association between danger signs and recovery rate. Two studies' findings [28, 37] indicated a significant association, and the findings of one study indicated no significant association [38]. The results of this meta‐analysis revealed that children with danger signs had a lower recovery rate from pneumonia than children without danger signs (AHR = 0.78, 95% CI: 0.66–0.9) with no heterogeneity (Figure 9).

### 5.6 The Association Between Lack of Oxygen and Recovery Rate

**Figure 10:**
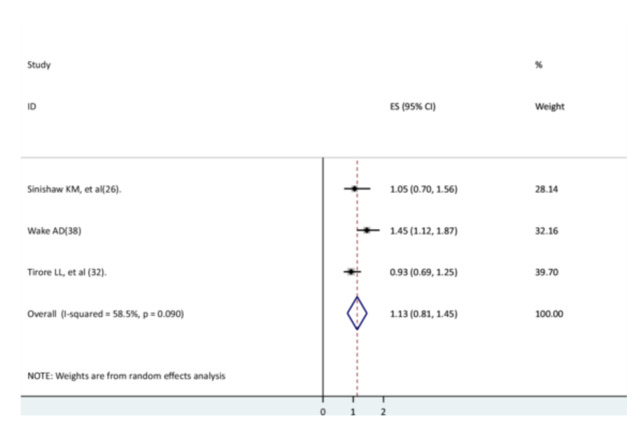
The association between lack of oxygen and the recovery rate of children from pneumonia.

Three studies [26, 32, 33] were used to assess children with and without oxygen recovery from pneumonia. Two studies' findings [26, 33] did not show a significant association, but the other study had a significant association [32]. This systematic review and meta‐analysis indicated that the recovery rate of children from pneumonia did not differ significantly between those who received oxygen and those who did not receive oxygen (AHR = 1.13, 95% CI: 0.81–1.45). There is moderate heterogeneity between studies (Figure 10).

### 5.7 The Association Between Vaccination Status and Recovery of Children From Pneumonia

**Figure 11:**
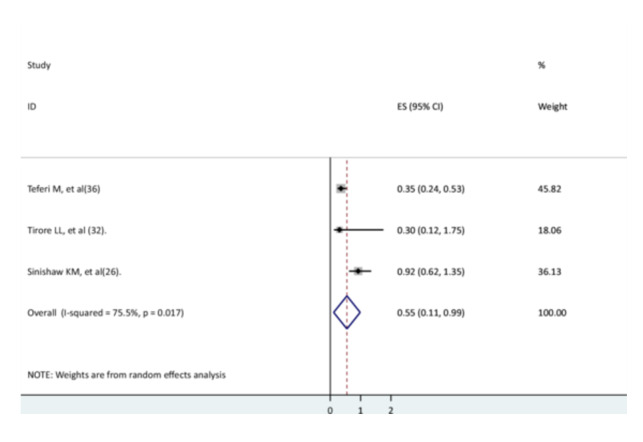
The association between the vaccination status of children and their recovery from pneumonia.

Three studies were used to investigate the relationship between children's pneumonia recovery rate and vaccination status [26, 31, 33]. With the exception of one study [31], the findings of two studies [26, 33] showed no significant association. The result of the meta‐analysis revealed that children who were not fully vaccinated had a 45% lower recovery rate than children who were fully vaccinated (AHR = 0.55, 95% CI: 0.11–0.99) with high heterogeneity (Figure 11).

### 5.8 The Association Between the Presence of Comorbidity and the Recovery of Children From Pneumonia

**Figure 12:**
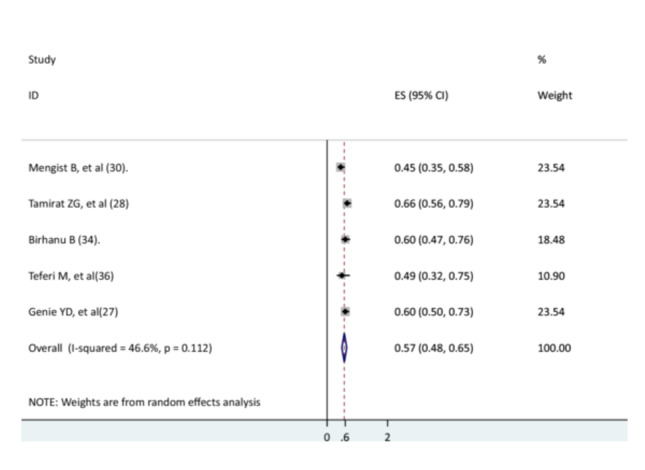
The association between comorbidity and recovery of children from pneumonia.

The association between comorbidity and recovery of children from pneumonia was assessed using five studies [27, 28, 31, 37, 38]. The findings of all studies revealed that comorbidity reduced the recovery rate of children from pneumonia. In the current meta‐analysis, children with comorbidity had a lower recovery rate from pneumonia than children without comorbidity (AHR = 0.57, 95% CI: 0.48–0.65) (Figure 12).

### 5.9 The Association Between History of Respiratory Tract Infection and Recovery of Children From Pneumonia

**Figure 13:**
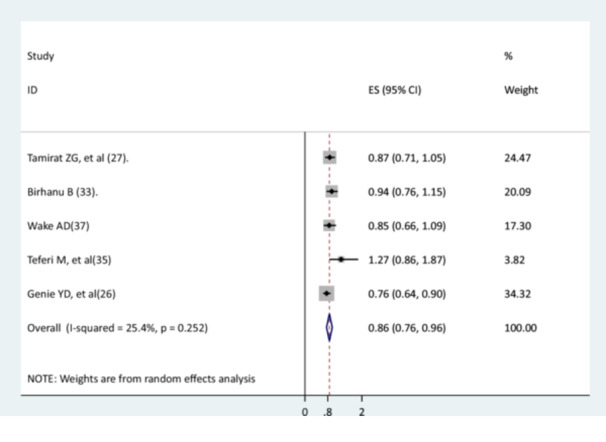
The relationship between the history of respiratory tract infection and recovery of children from pneumonia.

The relationship between the history of respiratory tract infection and recovery of children from pneumonia was investigated using five studies [26, 27, 30, 34, 36]. Four studies [27, 30, 34, 36] did not have significant associations, but one study had a significant association [26]. The findings of the meta‐analysis indicated that children who had a history of respiratory tract infections had a lower recovery rate than children without a history of respiratory tract infection (AHR = 0.86, 95% CI: 0.76–0.96). There was low heterogeneity between studies (Figure 13).

## 6 Discussion

To the best of our knowledge, there are no previous systematic reviews and meta‐analyses that have examined the national estimate of the recovery rate and its determinants among children with pneumonia. The current systematic review and meta‐analysis included cohort studies published in Ethiopia and reported the pooled incidence rate of the recovery of children from pneumonia and the risk factors that affected it.

In this systematic review and meta‐analysis, the pooled recovery rate of children from pneumonia was 17.7 (95% CI: 14.61–20.79) per 100 children per day. Despite the efforts implemented in medical facilities in Ethiopia to improve the prognosis of children with pneumonia, the recovery rate of children with pneumonia is still very low. This showed that pneumonia is the primary cause of death for children worldwide [2, 3], despite low mortality rates in developed countries [4]. In the current study, the recovery rate of children from pneumonia was 17.7 per 100 children per day. Conversely, findings from a Nepal study showed that children with severe pneumonia had a median recovery time of 49 h, indicating that 50% had recovered within just over 2 days [5]. A previous district‐level study conducted in Jimma reported that 83.91% of children recovered from severe pneumonia, providing a cumulative proportion of recovery over the study period [7]. In contrast, the current meta‐analysis conducted across Ethiopia reported a recovery rate of 17.7 per 100 children per day, which reflects the incidence rate of recovery—that is, the speed at which recovery occurred. This indicates that caution must be taken when comparing a cumulative recovery proportion with a daily recovery rate, as they represent different aspects of disease progression and treatment outcomes. The current study's findings on the delayed recovery rate of children from pneumonia could be attributed to inadequate immunization and service usage improvements [9], inadequate facility preparation, and an absence of high‐quality care [10]. The change in etiological profile of pneumonia can also influence the recovery rate [8]. The delayed recovery rate may also be due to very sick children presenting at the hospitals after failed alternative care. However, under the assumption of a constant recovery rate and no competing risks, the current recovery rate was relatively faster than that reported in a previous Ethiopian study, which reported a median recovery time of 4 days [6]. Sample size, where the current study had a larger sample size, in which the outcome was detected in a more precise way, and clinical characteristics among study participants could be the causes of the variation in the recovery rate of children.

The highest recovery rate of children from pneumonia was observed in the southern nation, at a rate of 20 per 100 children per day, while the lowest recovery rate was in Addis Ababa, at 12 per 100 children per day. The disparities in treatment accessibility [11, 14] and the occurrence of comorbidity and malnourishment [16, 17, 18] could be the cause of the regional differences in recovery rates. Another explanation could be attributed to the variation in improved housing quality and early childhood nutrition [15]. In a retrospective study design, the recovery rate of children from pneumonia was 19 per 100 children per day; in a prospective study design, the recovery rate was 14 per 100 children per day. The reasons may be that including many studies resulted in a larger sample size in retrospective study designs, which helps in properly detecting the outcome compared to including a few studies in prospective study designs. For median follow‐up periods longer than 4 days, the recovery rate of children from pneumonia was 12 per 100 children per day; for median follow‐up periods shorter than 4 days, the recovery rate was 21 per 100 children per day. Malnutrition, comorbidities, and infections may be causes for children whose median follow‐up period exceeds 4 days [16, 17, 18].

The recovery rate of rural children from pneumonia was slower than that of children with urban residence. Poor household sanitation may be a risk for infection, which in turn causes failure of recovery at the hospital [15] and inadequate access to healthcare in rural areas [14]. Stunting children had a low recovery rate from pneumonia compared to normal children. This evidence has consistent findings with a previous study's findings that found that stunting prolonged the duration of recovery in children with pneumonia [17]; it is also in line with another study that reported that the risk of death in children with pneumonia increased with the severity of malnutrition [16]. Children with danger signs had a lower recovery rate from pneumonia than those without danger signs. Thus, healthcare providers should carefully screen dander signs [12], which aids in timely treatment of children, that is essential for children's survival [13]. Children with comorbidities and histories of respiratory infections had a slower recovery rate than children without comorbidities and histories of respiratory infections. Children who did not receive full vaccination were less likely to recover from pneumonia than those who received full vaccination. This evidence is in line with a previous study that reported that risk factors for host‐related infection are comorbidities, respiratory infection, and incomplete series vaccinations [18].

Most of the studies in this systematic review and meta‐analysis were retrospective, which depended on records, making it impossible to control confounding factors by including relevant variables such as maternal sociodemographic, socioeconomic, and environmental factors. The presence of significant heterogeneity across the studies may be caused by clinical differences between study subjects and study methodology differences among studies. The presence of publication bias indicated that there was a small study effect on the overall pooled incidence rate. Few regions in Ethiopia were represented in this study. A few of the subgroup analyses had one, two, or three studies with a small sample size, which made it difficult to detect the outcome accurately. Even though this study had shortcomings, it is the first in Ethiopia to determine the recovery rate of children from pneumonia and its predictors. Health offices and programmers use this information to improve the clinical service management of health institutions in Ethiopia.

## 7 Conclusion

In the current study, the recovery rate of children from pneumonia was 17.7 per 100 child‐days. Children who lived in rural areas recovered from pneumonia more slowly. The health care provider should be able to screen and closely follow up for children with stunted, danger signs, comorbid, and a history of respiratory infection at admission. The quality of diagnosis and management of pneumonia at primary care levels, which are often the first points of contact of patients with their health systems, should be strengthened. Achieving complete immunization status should improve the recovery rate.

## Author Contributions

**Temesgen Gebeyehu Wondmeneh:** conceptualization, methodology, software, formal analysis, project administration, resources, supervision, validation, visualization, writing – review and editing, investigation, funding acquisition, writing – original draft, and data curation. **Abduhakm Hara Hadato:** conceptualization, investigation, funding acquisition, writing – original draft, writing – review and editing, visualization, methodology, validation, software, formal analysis, project administration, data curation, supervision, and resources.

## Conflicts of Interest

The authors declare no conflicts of interest.

## Transparency Statement

The authors, Temesgen Gebeyehu Wondmeneh and Abdulhakim Hora Hadato, affirm that this manuscript is an honest, accurate, and transparent account of the study being reported; that no important aspects of the study have been omitted; and that any discrepancies from the study as planned (and, if relevant, registered) have been explained.

## Supporting information

S1 File.

S2 File.

S3 File.

S4 File.

## Data Availability

All data generated or analyzed during this study are included in this article.

## References

[1] “Pneumonia in Children,” UNICEF, published November 10, 2023, https://www.unicef.org/stories/childhood-pneumonia-explained.

[2] “Pneumonia in Children,” WHO, published November 11, 2022, https://www.who.int/news-room/fact-sheets/detail/pneumonia.

[3] R. G. Bender , S. B. Sirota , L. R. Swetschinski , et al., “Global, Regional, and National Incidence and Mortality Burden of Non‐COVID‐19 Lower Respiratory Infections and Aetiologies, 1990–2021: A Systematic Analysis From the Global Burden of Disease Study 2021,” Lancet Infectious Diseases 24, no. 9 (2024): 974–1002. 38636536 10.1016/S1473-3099(24)00176-2 PMC11339187

[4] A. Meliyanti , D. Rusmawatiningtyas , F. Makrufardi , and E. Arguni , “Factors Associated With Mortality in Pediatric Pneumonia Patients Supported With Mechanical Ventilation in Developing Country,” Heliyon 7, no. 5 (2021): e07063. 34041404 10.1016/j.heliyon.2021.e07063 PMC8141870

[5] S. Basnet , A. Sharma , M. Mathisen , et al., “Predictors of Duration and Treatment Failure of Severe Pneumonia in Hospitalized Young Nepalese Children,” PLoS One 10, no. 3 (2015): e0122052. 25798907 10.1371/journal.pone.0122052 PMC4370861

[6] R. A. Amare , G. Fisseha , A. Berhe , and L. L. Tirore , “Incidence of Recovery From Severe Pneumonia and Its Predictors Among Children 2–59 Months Admitted to Pediatric Ward of Ayder Comprehensive Specialized Hospital, Tigray, Ethiopia: A Retrospective Cohort Study,” Journal of Family Medicine and Primary Care 11, no. 9 (2022): 5285–5292. 10.4103/jfmpc.jfmpc_2006_21 PMC9731067 36505606

[7] K. Tegenu , G. Geleto , D. Tilahun , E. Bayana , and B. Bereke , “Severe Pneumonia: Treatment Outcome and Its Determinant Factors Among Under‐Five Patients, Jimma, Ethiopia,” SAGE Open Medicine 10 (2022): 20503121221078445. 35223030 10.1177/20503121221078445 PMC8873968

[8] S. Khadanga , T. Karuna , P. Thatoi , and S. Behera , “Changing Bacteriological Profile and Mortality Trends in Community Acquired Pneumonia,” Journal of Global Infectious Diseases 6, no. 4 (2014): 186–188. 25538458 10.4103/0974-777X.145251 PMC4265835

[9] A. Tariku , Y. B. Okwaraji , A. Worku , G. A. Biks , L. Åke Persson , and Y. Berhane , “Prevention and Treatment of Suspected Pneumonia in Ethiopian Children Less Than Five Years From Household to Primary Care,” Acta Paediatrica 110, no. 2 (2021): 602–610. 32478446 10.1111/apa.15380 PMC7891650

[10] A. Tariku , Y. Berhane , A. Worku , G. A. Biks , L. Å. Persson , and Y. B. Okwaraji , “Health Postservice Readiness and Use of Preventive and Curative Services for Suspected Childhood Pneumonia in Ethiopia: A Cross‐Sectional Study,” BMJ Open 12, no. 4 (2022): e058055. 10.1136/bmjopen-2021-058055 PMC9047705 35477882

[11] F. Ferdous , S. Ahmed , S. K. Das , et al., “Pneumonia Mortality and Healthcare Utilization in Young Children in Rural Bangladesh: A Prospective Verbal Autopsy Study,” Tropical Medicine and Health 46, no. 1 (2018): 17. 29875615 10.1186/s41182-018-0099-4 PMC5970515

[12] Y. Jahan , S. A. Rahman , A. S. Chowdhury , and M. Moshiur Rahman , “Management of Severe Childhood Pneumonia by Day Care Approach in Developing Countries,” Health Promotion Perspectives 8, no. 1 (2018): 88–91. 29423367 10.15171/hpp.2018.11 PMC5797313

[13] I. K. Ndu , U. Ekwochi , C. D. I. Osuorah , et al., “Danger Signs of Childhood Pneumonia: Caregiver Awareness and Care Seeking Behavior in a Developing Country,” International Journal of Pediatrics 2015 (2015): 167261. 26576161 10.1155/2015/167261 PMC4632178

[14] S. Graham , “Challenges to Improving Case Management of Childhood Pneumonia at Health Facilities in Resource‐Limited Settings,” Bulletin of the World Health Organization 86, no. 5 (2008): 349–355. 18545737 10.2471/BLT.07.048512 PMC2647436

[15] C. C. Grant , D. Emery , T. Milne , et al., “Risk Factors for Community‐Acquired Pneumonia in Pre‐School‐Aged Children,” Journal of Paediatrics and Child Health 48, no. 5 (2012): 402–412. 22085309 10.1111/j.1440-1754.2011.02244.x

[16] A. Kirolos , R. M. Blacow , A. Parajuli , et al., “The Impact of Childhood Malnutrition on Mortality From Pneumonia: A Systematic Review and Network Meta‐Analysis,” BMJ Global Health 6, no. 11 (2021): e007411. 10.1136/bmjgh-2021-007411 PMC8634228 34848440

[17] P. P. Moschovis , E. O. Addo‐Yobo , S. Banajeh , et al., “Stunting Is Associated With Poor Outcomes in Childhood Pneumonia,” Tropical Medicine & International Health: TM & IH 20, no. 10 (2015): 1320–1328. 26083963 10.1111/tmi.12557 PMC4729453

[18] R. B. Aurilio , C. C. Sant'Anna , and M. F. B. P. March , “Clinical Profile of Children With and Without Comorbidities Hospitalized With Community‐Acquired Pneumonia,” Revista Paulista de Pediatria 38 (2020): e2018333. 32401948 10.1590/1984-0462/2020/38/2018333 PMC7212558

[19] M. J. Page , J. E. McKenzie , P. M. Bossuyt , et al., “The PRISMA 2020 Statement: An Updated Guideline for Reporting Systematic Reviews,” PLOS Medicine 18, no. 3(2021): e1003583, 33780438 10.1371/journal.pmed.1003583 PMC8007028

[20] G. A Wells , B. Shea , D. O'Connell , et al., “The Newcastle‐Ottawa Scale (NOS) for Assessing the Quality of Nonrandomised Studies in Meta‐Analyses,” published 2014, https://www.ohri.ca/programs/clinical_epidemiology/oxford.asp.

[21] R. DerSimonian and R. Kacker , “Random‐Effects Model for Meta‐Analysis of Clinical Trials: An Update,” Contemporary Clinical Trials 28, no. 2 (2007): 105–114. 16807131 10.1016/j.cct.2006.04.004

[22] H. Israel and R. R. Richter , “A Guide to Understanding Meta‐Analysis,” Journal of Orthopaedic and Sports Physical Therapy 41, no. 7 (2011): 496–504, https://www.jospt.org/doi/epdfplus/10.2519/jospt.2011.3333;. 21725192 10.2519/jospt.2011.3333

[23] M. Egger , G. D. Smith , M. Schneider , and C. Minder , “Bias in Meta‐Analysis Detected by a Simple, Graphical Test,” BMJ 315, no. 7109 (1997): 629–634. 9310563 10.1136/bmj.315.7109.629 PMC2127453

[24] J. A. C. Sterne and M. Egger , “Funnel Plots for Detecting Bias in Meta‐Analysis,” Journal of Clinical Epidemiology 54, no. 10 (2001): 1046–1055. 11576817 10.1016/s0895-4356(01)00377-8

[25] M. B. Mathur and T. J. VanderWeele , “Sensitivity Analysis for Publication Bias in Meta‐Analyses,” Journal of the Royal Statistical Society Series C: Applied Statistics 69, no. 5 (2020): 1091–1119. 33132447 10.1111/rssc.12440 PMC7590147

[26] K. M. Sinishaw , G. Sebsbie , and M. A. Kebede , “Predictors of Recovery Time From Severe Community‐Acquired Pneumonia Among Paediatrics Patients in Selected Hospitals in Addis Ababa, Ethiopia: An Institution‐Based Retrospective Cohort Study,” BMJ Open 14, no. 3 (2024): e078721. 10.1136/bmjopen-2023-078721 PMC10961574 38514151

[27] Y. D. Genie , A. Sayih , N. Dessalegn , et al., “Time to Recovery From Severe Pneumonia and Its Predictors Among Pediatric Patients Admitted in South West Region Governmental Hospitals, South West Ethiopia: Prospective Follow‐Up Study,” Global Pediatrics 9 (2024): 100227.

[28] Z. G. Tamirat , A. A. Gelagay , and M. M. Boke , “Time to Recovery And Its Predictors Among Children Aged 2 to 59 Months Admitted With Severe Community‐Acquired Pneumonia in Public Hospitals of Central and North Gondar Zones, Ethiopia 2021,” Research Square [Preprint] 2022, https://assets.researchsquare.com/files/rs-1850638/v1/c2f6cc67-9da7-49e5-93f1-ed6912ca5381.pdf?c=1661157791.

[29] A. Kassaw , G. Kerebih , S. Zeleke , et al., “Survival Status and Predictors of Mortality From Severe Community‐Acquired Pneumonia Among Under‐Five Children Admitted at Debre Tabor Comprehensive Specialized Hospital: A Prospective Cohort Study,” Frontiers in Pediatrics 11 (2023): 1141366. 37346893 10.3389/fped.2023.1141366 PMC10280987

[30] I. R. Dinka , D. Seyoum , S. Debelo , et al., “Time to Recovery and Its Predictors Among Under‐Five Children Admitted With Severe Pneumonia in East Wallaga Zone Public Hospitals, Western Ethiopia, 2023; a Retrospective Cohort Study,” BMC Pediatrics 24, no. 1 (2024): 459. 39026278 10.1186/s12887-024-04937-2 PMC11256476

[31] M. Teferi , E. Addisu , S. Wodajo , et al., “Time to Recovery From Severe Community‐Acquired Pneumonia and Its Predictors Among 6 to 59 Months of Age Children Admitted to South Wollo Zone Public Hospitals, North East Ethiopia: A Prospective Follow‐Up Study,” Pneumonia 16, no. 1 (2024): 14. 39098940 10.1186/s41479-024-00135-x PMC11299310

[32] A. D. Wake , “Recovery Time From Severe Community Acquired Pneumonia and Risk Factors Among Pediatrics, Ethiopia: A Retrospective Follow‐Up Study,” Global Pediatric Health 11 (2024): 2333794x241256860. 10.1177/2333794X241256860 PMC11177736 38882550

[33] L. L. Tirore , D. E. Abame , T. Sedoro , et al., “Time to Recovery From Severe Pneumonia and Its Predictors Among Children 2–59 Months of Age Admitted to Pediatric Ward of Nigist Eleni Mohammed Memorial Comprehensive Specialized Hospital, Hossana, Ethiopia: Retrospective Cohort Study,” Pediatric Health, Medicine and Therapeutics 12 (2021): 347–357. 34321951 10.2147/PHMT.S321184 PMC8312316

[34] B. Fenta Kebede , A. Yetwale Hiwot , T. Biyazin Tesfa , Y. Dagnaw Genie , and N. Dessalegn Mulu , “Time to Recovery From Severe Pneumonia and Its Predictors Among Pediatric Patients Admitted in Mizan‐Tepi University Teaching Hospital, South West Ethiopia, 2022,” Frontiers in Nursing 3 (2024): 343–354, https://intapi.sciendo.com/pdf/10.2478/fon-2024-0038.

[35] H. Dinku , D. Amare , S. Mulatu , and M. D. Abate , “Predictors of Prolonged Hospitalization Among Children Aged 2–59 Months With Severe Community‐Acquired Pneumonia in Public Hospitals of Benishangul‐Gumuz Region, Ethiopia: A Multicenter Retrospective Follow‐Up Study,” Frontiers in Pediatrics 11 (2023): 1189155. 37484762 10.3389/fped.2023.1189155 PMC10357288

[36] T. Assfaw , C. Yenew , K. Alemu , W. Sisay , and T. Geletaw , “Time‐To‐Recovery From Severe Pneumonia and Its Determinants Among Children Under‐Five Admitted to University of Gondar Comprehensive Specialized Hospital in Ethiopia: A Retrospective Follow‐Up Study; 2015–2020,” Pediatric Health, Medicine and Therapeutics 12 (2021): 189–196. 33907491 10.2147/PHMT.S305383 PMC8071205

[37] B. Mengist , M. Tesfa , and B. Kassie , “Time to Recovery and Predictors of Severe Community‐Acquired Pneumonia Among Pediatric Patients in Debre Markos Referral Hospital, North West Ethiopia: A Retrospective Follow‐Up Study,” PLoS One 15, no. 9 (2020): e0239655. 32976491 10.1371/journal.pone.0239655 PMC7518609

[38] B. Bethelhem , “Time to Recovery and its Predictors of Severe Community‐Acquired Pneumonia Among Children 1‐59 Months of Age Admitted in Paediatric Ward of North Shoa Public Hospitals, Amhara region, Ethiopia, 2022” (thesis, Debre Berhan University, 2022), https://etd.dbu.edu.et/bitstream/handle/123456789/1196/Bethelhem%20Birhanu%20research%20on%20%20time%20to%20%20recovery%20of%20%20SCAP.pdf?sequence=1&isAllowed=y.

