# Supplementary material for: Recovery Rate of Children From Pneumonia and Its Predictors in Ethiopia: A Systematic Review and Meta‐Analysis
Source: Health Sci Rep. 2025 Sep 1;8(9):e71127. doi: 10.1002/hsr2.71127 (PMC12399988; doi:10.1002/hsr2.71127)
Supplement: Supplementary file 2 — S2 File. [file HSR2-8-e71127-s003.docx]

S2 File. Comprehensive search strategy for recovery rate of children from pneumonia in Ethiopia

| Databases | Key search terms or phrases |
| --- | --- |
| **Web of science** | Recovery rate AND children AND Pneumonia AND Ethiopia |
| Search Filters | any field, contains exact phrase, articles and English language |
| Total articles | 58 |
| **Science direct** | "Recovery rate" AND children AND Pneumonia AND Ethiopia |
| Limited by | research articles and open access |
| Total articles | 16 |
| **PubMed** | ((("Recovery rate") AND ("children")) AND ("Pneumonia")) AND ("Ethiopia") |
| Limited by | free full text |
| Total articles | 10 |
| **Google scholar** | "Recovery rate" children pneumonia Ethiopia |
| Total articles | 67 |
| **African Journals Online** | "Recovery rate" children pneumonia Ethiopia |
| Total articles | 9 |
| **Ethiopian higher institution repository** | Recovery rate of children from pneumonia in Ethiopia |
| Total articles | 2 |
| **Total articles retrieved from all** **databases** | **162** |
