# Supplementary material for: Recovery Rate of Children From Pneumonia and Its Predictors in Ethiopia: A Systematic Review and Meta‐Analysis
Source: Health Sci Rep. 2025 Sep 1;8(9):e71127. doi: 10.1002/hsr2.71127 (PMC12399988; doi:10.1002/hsr2.71127)
Supplement: Supplementary file 3 — S3 File. [file HSR2-8-e71127-s004.docx]

| Authors | publication year | study year | study region | study design | sample size | cases | person time | Incidence rate | response rate |
| --- | --- | --- | --- | --- | --- | --- | --- | --- | --- |
| Kassaw A, et al (29). | 2023 | 2021-2022 | Amhara | prospective | 580 | 451 | 4716 child-days | 9.6 per 100 child-day | 98.30% |
| Mengist B,et al (30). | 2020 | 2016-2018 | Amhara | retrospective | 352 | 313 | 1923 child-days | 16.25 per 100 person-days | 100% |
| Dinku H, et al (31). | 2021 | 2016-2020 | Beshangul Gumuz | retrospective | 515 | 488 | 2478 person-days | 19.69per 100 person-days | 97.90% |
| Sinishaw KM, et al (26). | 2024 | 2018-2020 | Addis Ababa | retrospective | 388 | 11.5 | 100 person-day | 11.5 per 100 person-day | 95.50% |
| Tirore LL, et al (32) | 2021 | 2017-2020 | SNNP | retrospective | 280 | 260 | 1076 child- days | 24.16 per 100 person-days | 100% |
| Assfaw T, et al (33). | 2021 | 2015-2020 | Amhara | retrospective | 330 | 120 | 889 child-days | 13.5 per per 100-persons | 100% |
| Tamirat ZG, et al (28). | 2022 | 2018-2020 | Amhara | retrospective | 701 | 688 | 2567 child-days | 26.71 per 100 person-days | 100% |
| Birhanu B (34). | 2022 | 2019-2021 | Amhara | retrospective | 587 | 427 | 2863 child-days | 14.53 per 100 person-days | 95.4% |
| Genie YD, et al (27) | 2024 | 2022 | SNNP | prospective | 791 | 641 | 5134 child-days | 12.5 per 100 person-days | 99% |
| Dinka I.R, et al(35) | 2024 | 2017-2022 | Oromia | retrospective | 376 | 356 | 1599 child-days | 22.26 per 100 person-days | 98% |
| Teferi M, et al(36) | 2024 | 2021 | Amhara | prospective | 270 | 205 | 1002 child-days | 20.45 per 100 person-days | 100% |
| Kebede B F,et al(37) | 2024 | 2019-2021 | SNNP | retrospective | 591 | 541 | 2245 child-days | 24.1 per 1000 person-days | 97% |
| Wake AD(38) | 2024 | 2021-2022 | Oromia | retrospective | 412 | 369 | 2819 child-days | 13.1 per 1000 person-days | 92% |

S3 File. Extracted data for recovery rate of children from pneumonia
